# Supplementary figures and images for: The Intratumor Bacterial and Fungal Microbiome Is Characterized by HPV, Smoking, and Alcohol Consumption in Head and Neck Squamous Cell Carcinoma
Source: Int J Mol Sci. 2022 Oct 31;23(21):13250. doi: 10.3390/ijms232113250 (PMC9655846; doi:10.3390/ijms232113250)

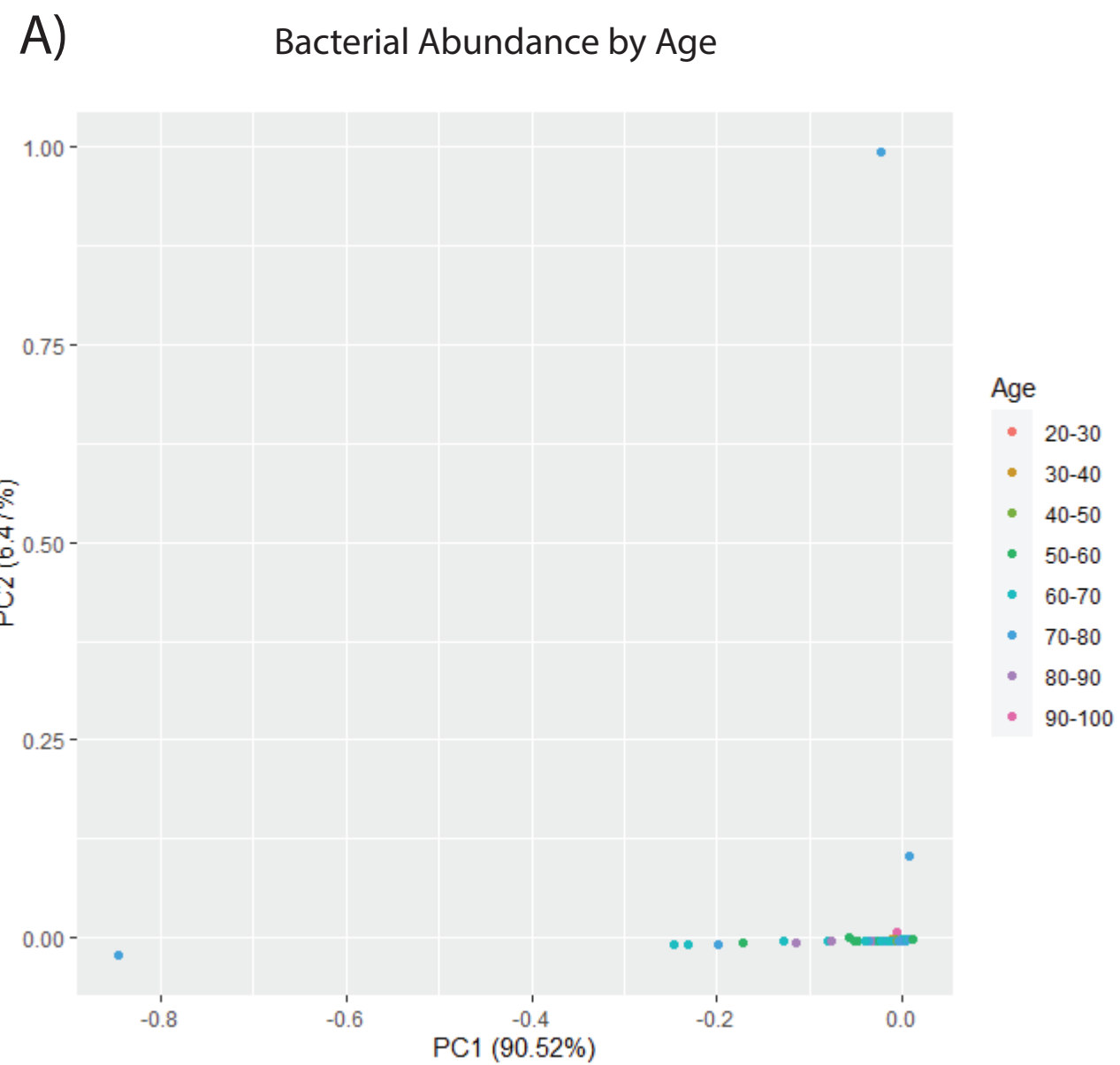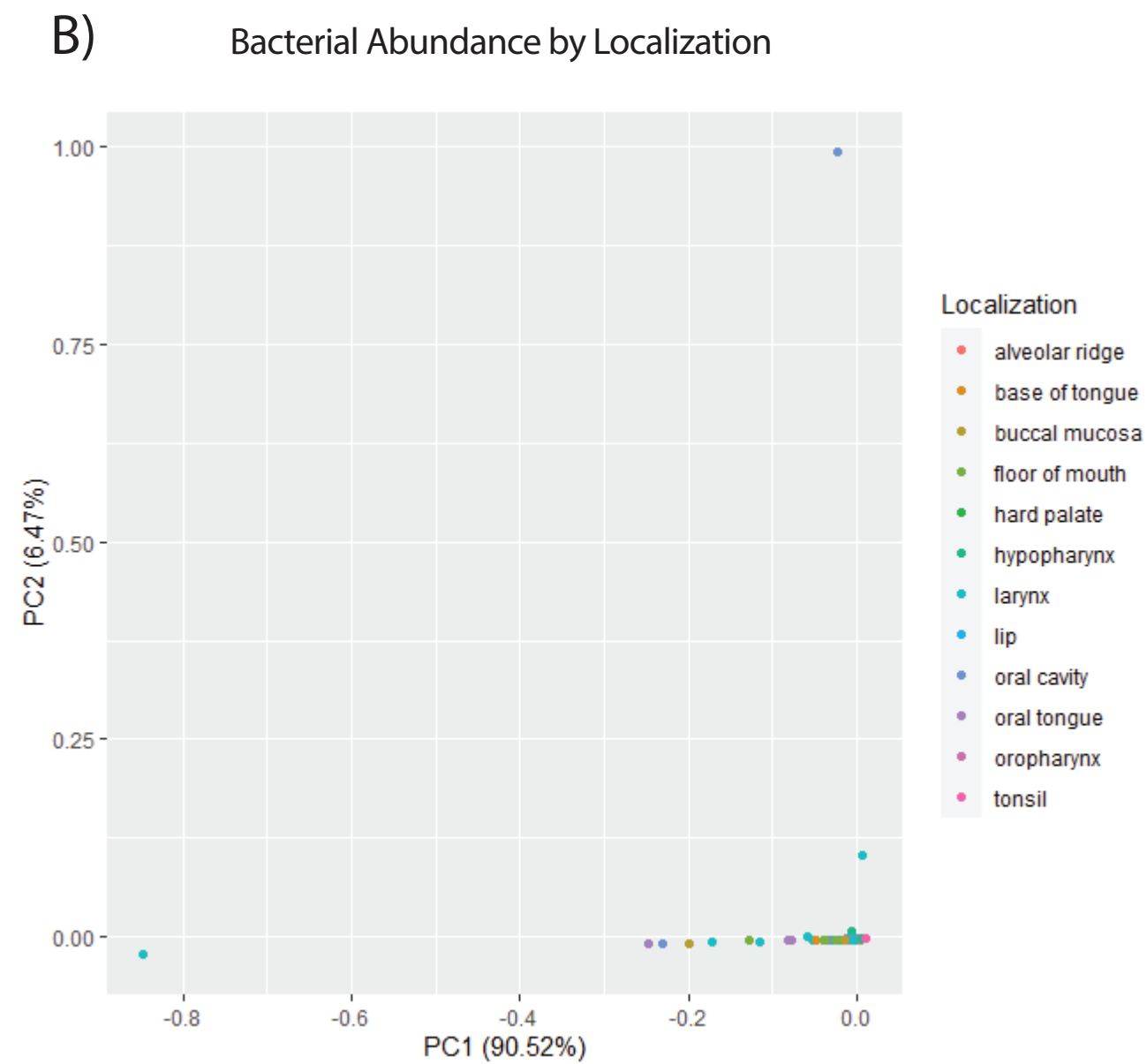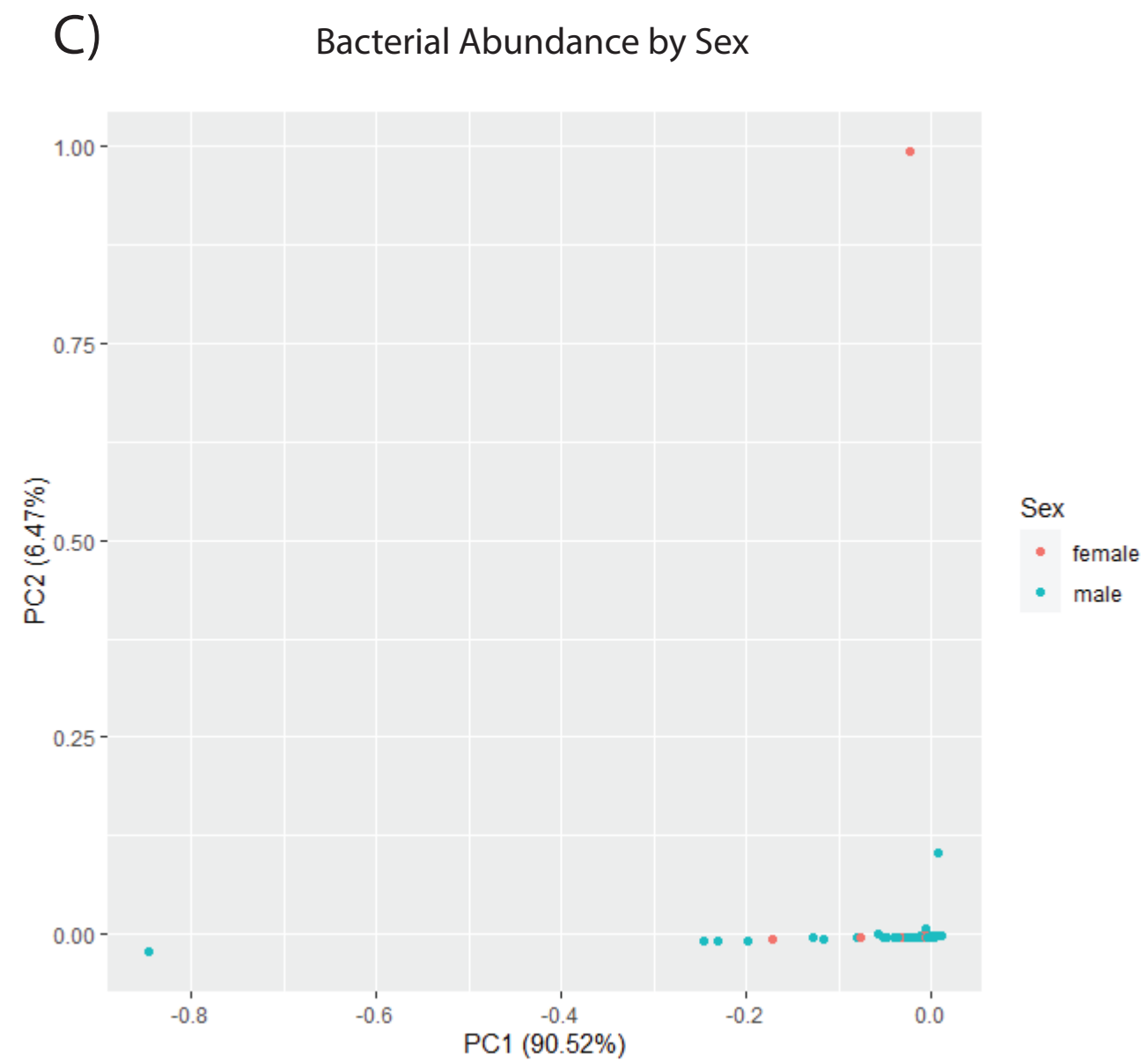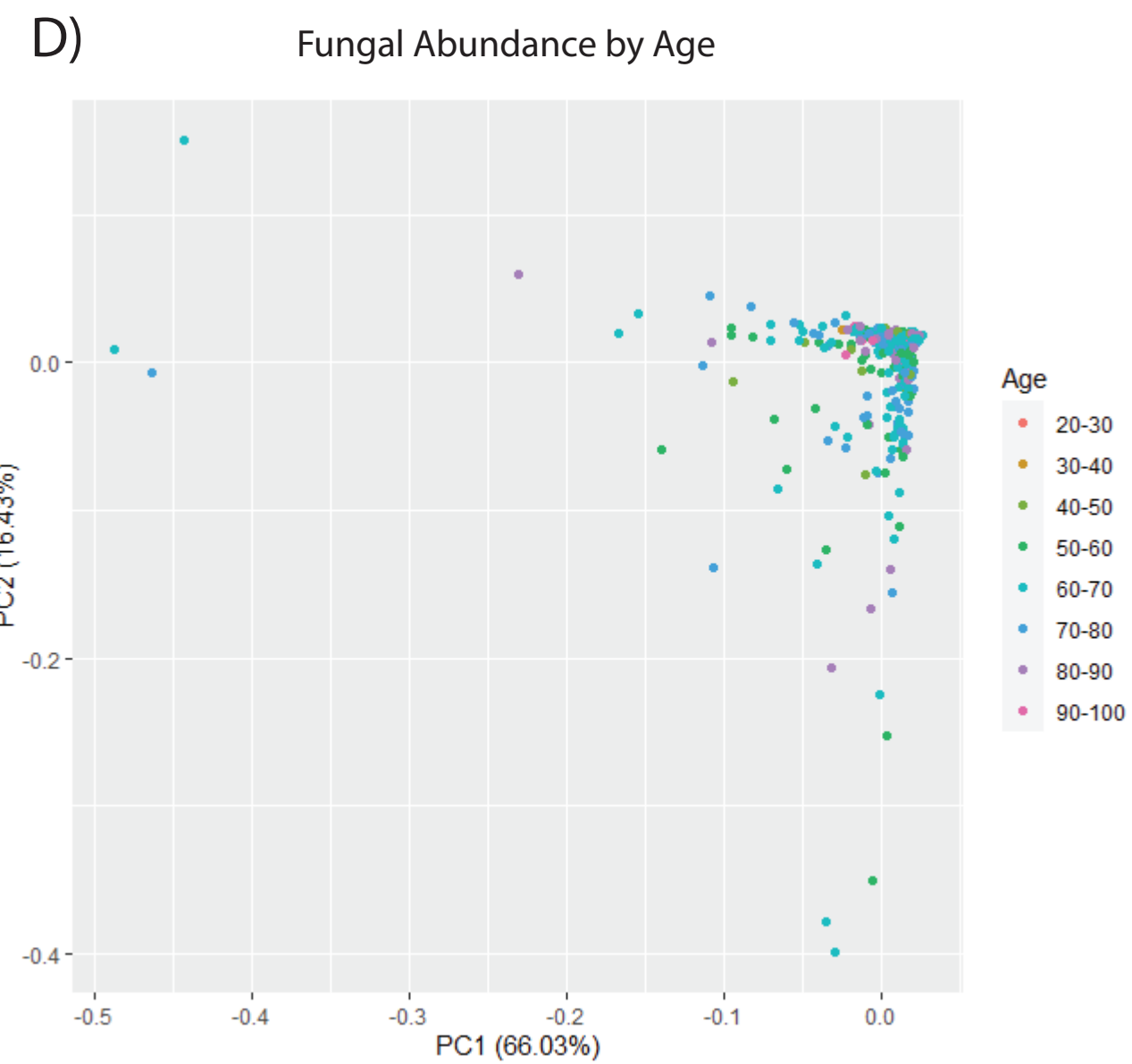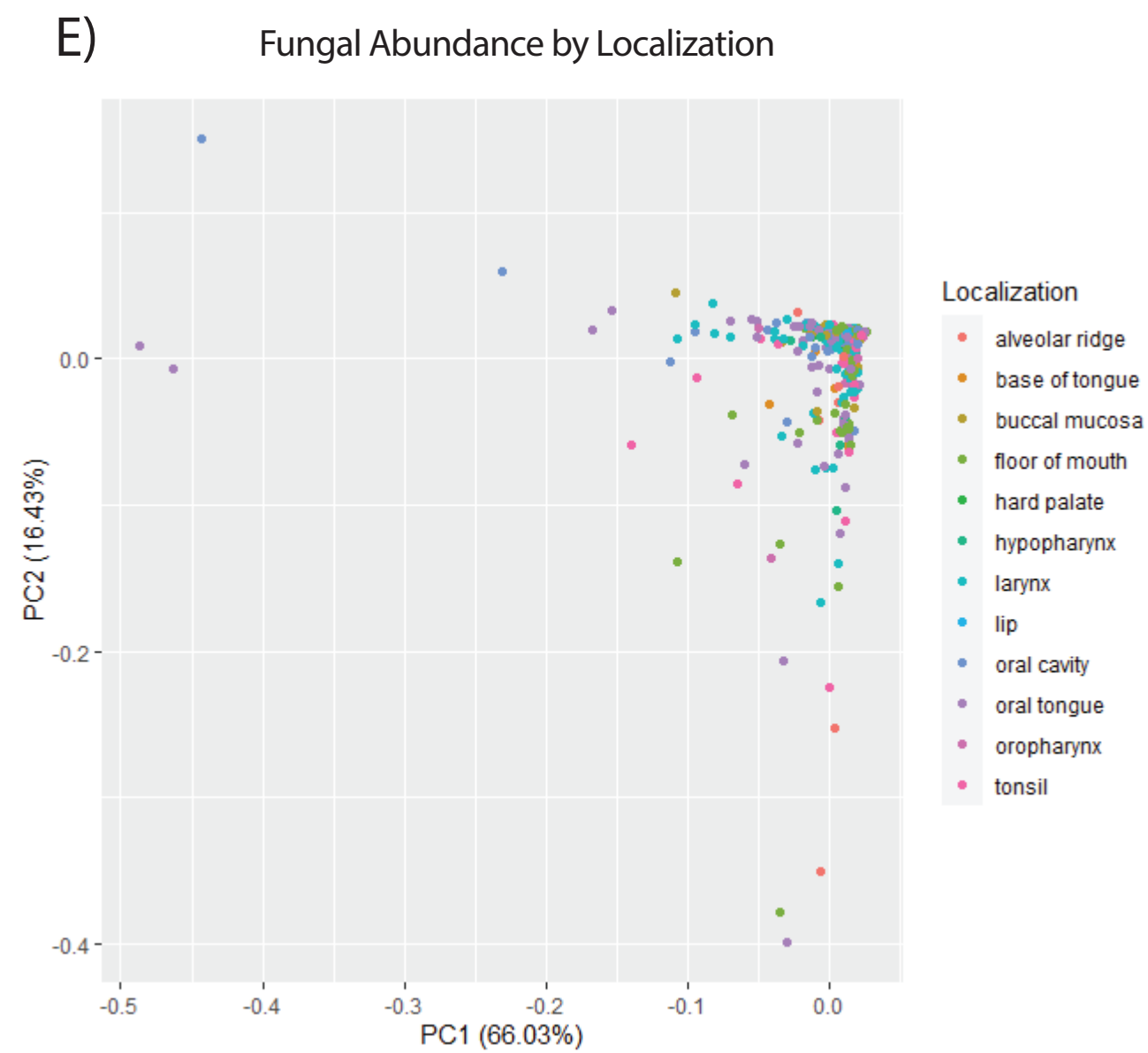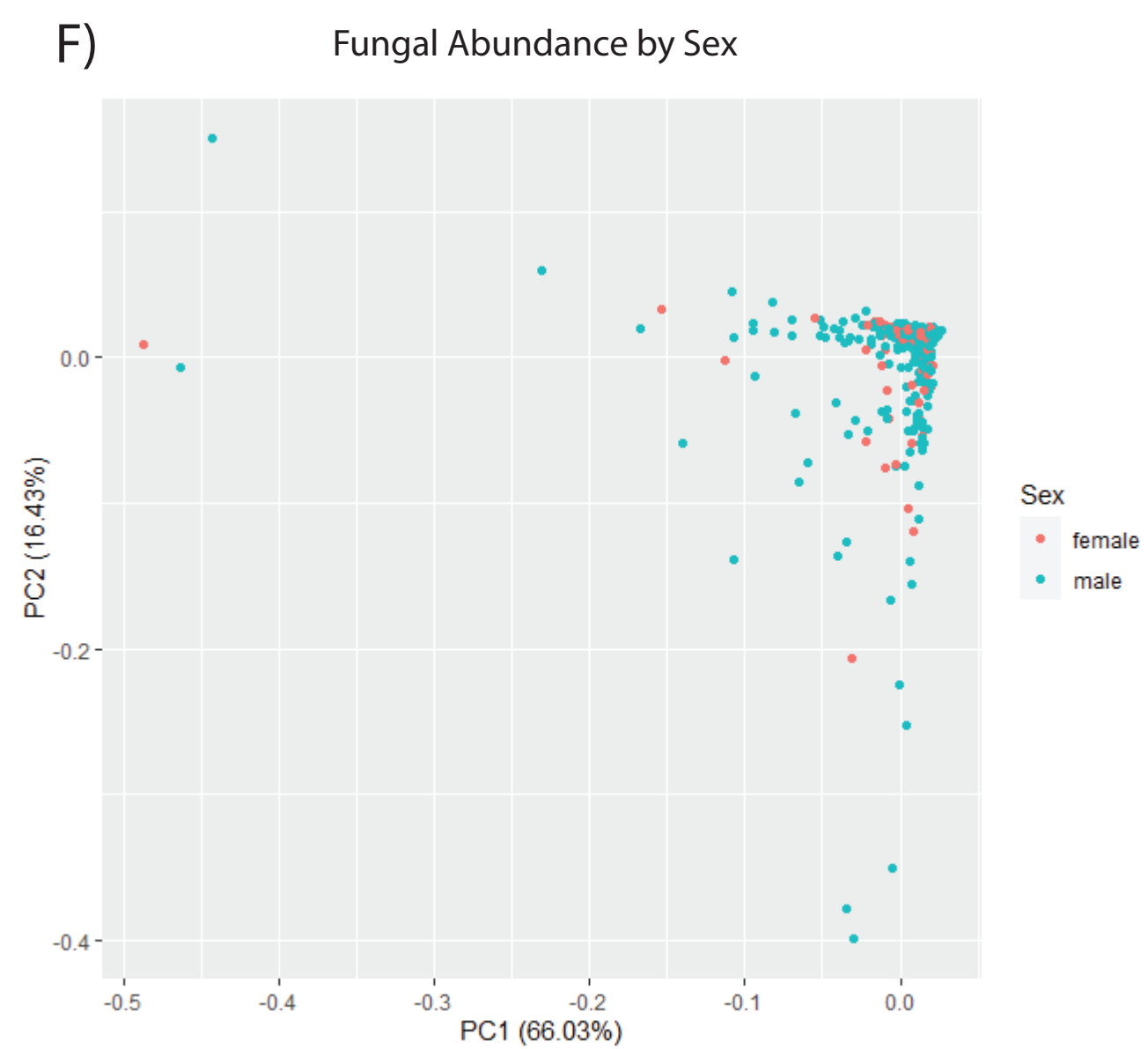

Supplement: Supplementary file 1 [file ijms-23-13250-s001.zip › Supplementary Figure S1.pdf]
